# Additive Hazard Regression Models: An Application to the Natural History of Human Papillomavirus

**DOI:** 10.1155/2013/796270

**Published:** 2013-01-28

**Authors:** Xianhong Xie, Howard D. Strickler, Xiaonan Xue

**Affiliations:** Department of Epidemiology and Population Health, Albert Einstein College of Medicine, Bronx, NY 10461, USA

## Abstract

There are several statistical methods for time-to-event analysis, among which is the Cox proportional hazards model that is most commonly used. However, when the absolute change in risk, instead of the risk ratio, is of primary interest or when the proportional hazard assumption for the Cox proportional hazards model is violated, an additive hazard regression model may be more appropriate. In this paper, we give an overview of this approach and then apply a semiparametric as well as a nonparametric additive model to a data set from a study of the natural history of human papillomavirus (HPV) in HIV-positive and HIV-negative women. The results from the semiparametric model indicated on average an additional 14 oncogenic HPV infections per 100 woman-years related to CD4 count < 200 relative to HIV-negative women, and those from the nonparametric additive model showed an additional 40 oncogenic HPV infections per 100 women over 5 years of followup, while the estimated hazard ratio in the Cox model was 3.82. Although the Cox model can provide a better understanding of the exposure disease association, the additive model is often more useful for public health planning and intervention.

## 1. Introduction

Time-to-event analysis is commonly used to study the risk factors associated with the incidence of clinical events [1]. For example, time-to-disease development, time-to-hospitalization, time-to-relapse/recurrence, and time-to-death are each frequently used as endpoints. However, there are several different models for measuring the relation of time-to-event data with risk factors, including parametric, semiparametric, and nonparametric models. In parametric models, a distribution is assumed for time to event (e.g., an exponential, gamma, or Weibull distribution), and it is further assumed that there is a linear relationship between the logarithm of time to event and the covariates in the model. The strength of association is then estimated using the maximum likelihood approach. In semiparametric models, most notably Cox proportional hazard regression models [2], the hazard function is assumed to be multiplicatively related to the covariates, with an unspecified baseline hazard function, and the maximum partial likelihood method is used to estimate the parameters. In nonparametric models, most notably the Kaplan-Meier approach, no assumptions are made regarding the relationship between the disease risk and the covariates. Instead, the survival function for each stratum of the covariates is estimated with empirical methods, and the log-rank test and other nonparametric tests are typically used to test the effects of these covariates.

A well-known but less often used method for analyzing time-to-event data is an additive hazard regression model [3]. Unlike the proportional hazards model which estimates hazard ratios, an additive model estimates the difference in hazards: the change in hazard function due to the exposure of interest or stated more simply the absolute difference in the instantaneous failure rate per unit of change in the exposure variable. Based on the estimate of difference in hazards, one can further estimate the change in cumulative incidence: when the cumulative hazard is small (e.g., rare events), the change in cumulative hazard approximates the difference in risk of disease due to exposure, that is, the attributable risk due to exposure. Therefore, when the attributable risk is of primary interest or the proportional hazard assumption is violated, an additive hazard regression model may be more appropriate. Since the nonparametric additive model was originally proposed by Aalen [3], there have been extensive researches on the topic [4–7]. However, the additive hazard regression models remain underutilized in public health and medical research primarily due to lack of familiarity with the models and lack of knowledge on how to implement the models using existing software. In this paper, we provide an example to illustrate the application of two additive models using existing statistical software (program codes are provided). 

The motivating example of this paper was a study of natural history of human papillomavirus (HPV) infection among human-immunodeficiency-virus- (HIV-) positive and HIV-negative women. The prior analysis of this data set used the Cox proportional hazard model to assess the relation of incident HPV detection with host immune status as measured by HIV serostatus and CD4 count [8, 9]. In this paper, we analyzed an updated version of this same data set with four years of additional followup, using additive hazards regression models to estimate the attributable risk of HPV infection related to changes in immune status and then contrasted these results with results using the Cox model. 

## 2. Methods

### 2.1. Data

The data were obtained from the Women's Interagency HIV Study (WIHS), a large ongoing multi-institutional observational study with semiannual clinical follow-up visits that include collection of exfoliated cervical cells for HPV DNA testing and Pap tests. There were 3766 women (2791 HIV+, 975 HIV−); two-thirds of whom were enrolled in 1994-95 and the remainder in 2001-02. Details of the study enrollment and methods have been previously reported [8, 9]. After excluding those women who had HIV seroconversion during followup, had hysterectomy prior to enrollment in WIHS, lacked HPV data during followup, or tested positive for oncogenic HPV at baseline, the number of women available for the current analysis of the incident detection of oncogenic HPV was 2386 (1672 HIV+, 714 HIV−). The oncogenic HPV types included HPV16, 18, 31, 33, 35, 39, 45, 51, 52, 56, 58, 59, and 68. We also studied the incident detection of any HPV in which more women were excluded because of being detected positive for any types of HPV at baseline; the corresponding number of women was 1733 (1116 HIV+, 617 HIV−). This data set represents an update from [8, 9] with 8 additional visits (4 additional years of followup).

Time-to-incident detection of HPV was estimated using midinterval between the last HPV-negative visit and the first HPV-positive visit. Time-to-incident detection of oncogenic or any HPV was analyzed separately. The primary exposure variable was host immune status characterized by HIV status and CD4 count: HIV-negative, HIV-positive with a CD4 count greater than 500, CD4 count between 200 and 500, and CD4 count less than 200. The additional covariates included age (<30, 30–34, 35–39, 40–44, ≥45 years), race (white, black, Hispanic, other), smoking (never, former, current), and the number of male sexual partner in past 6 months (0, 1, 2, ≥3).

### 2.2. Statistical Methods

Two additive hazard models were considered. The first model was the semiparametric additive hazard model

(1) $$\begin{array}{c} h\left(t\;|\;Z\right)=\beta_{0}\left(t\right)+\beta^{T}Z, \end{array}$$

where *h*(*t* | *Z*) is the conditional hazard rate of a given subject with the covariate *Z* = (*Z*_1_,…, *Z*_*p*_)^*T*^, *p* is the number of the covariates, *β*_0_(*t*) is the unknown baseline hazard function, and *β* = (*β*_1_,…, *β*_*p*_)^*T*^ is the unknown time-independent coefficients. In this study, we only considered the time-independent covariates, all at baseline. More general forms of the model (1) with time-dependent covariates have been studied in [4], which showed that the estimates of *β*_0_(*t*) and *β* are consistent and asymptotically normal. Note that the model (1) has a similar form to the Cox proportional hazard regression model: both models have an unspecified baseline hazard function and time-independent coefficients, although the Cox model is defined on a multiplicative scale while the additive hazard model is defined on an additive scale. Unlike the Cox proportional hazard regression model which requires numerical iterations in estimating the regression parameters, the previously mentioned semiparametric additive hazard regression model has closed form solution for estimating the regression parameters. We are able to estimate the absolute change in risk instead of relative change in risk with the model (1). The SAS code in [10] was used to fit the model, which produces the estimate for *β*, its standard error, and variance-covariance matrix. The *P* values were calculated under normal assumption. Additional SAS code was written to calculate the estimate of cumulative baseline hazard *B*_0_(*t*) = ∫_0_^*t*^ *β*_0_(*s*)*ds* and its standard errors based on [4]. The cumulative hazard function estimates were estimated based on the model (1). The Cox-Snell residual was evaluated for each subject at its observed survival time. Specifically, for a subject *i* with observed survival time *t*_*i*_, event indicator *δ*_*i*_ and covariate *z*_*i*_, the residual *r*_*i*_ is estimated by $\hat{B}{\;}_{0}(t_{i})+\hat{\beta }{\;\;}^{T}z_{i}t_{i}$. If the model (1) is correct, the *r*_*i*_'s should follow a unit exponential distribution with right censoring [1]. Because the unit exponential distribution has the property that its cumulative hazard function is the identity function, one can use this property to check the goodness of fit for the additive model. We therefore calculated the Nelson-Aalen estimates of cumulative hazards on the data (*r*_*i*_, *δ*_*i*_) for all subjects. In SAS, this can be obtained by using proc phreg with baseline statement and method=ch option in the statement (code is provided in online materials). The plot of the estimated cumulative hazards on the residuals *r*_*i*_ versus the residuals *r*_*i*_ was generated, in which a close to the 45 degree line is expected if the model (1) is true. 

Model (1) assumes that the effect of the covariate is constant on the hazard function, but in fact it can be generalized to any known parametric form that is possibly time dependent. We also considered a more general additive hazard model that allows the coefficients of the covariate to be time dependent and nonparametric,

(2) $$\begin{array}{c} h\left(t\;|\;Z\right)=\beta_{0}\left(t\right)+\beta {\left(t\right)}^{T}Z, \end{array}$$

where *β*(*t*) = (*β*_1_(*t*),…, *β*_*p*_(*t*))^*T*^. Unlike model (1), the new model makes no assumption regarding the form of *β*(*t*). The asymptotic theory of this model was studied in [11–13]. A SAS macro provided in [14] was used to fit the model, which gives the estimates of

(3) $$\begin{array}{c} B_{k}\left(t\right)=\int_{0}^{t}\beta_{k}\left(s\right)ds,\;k=0,\ldots ,p \end{array}$$

and their standard error estimates. The estimates for quantities *B*_*k*_(*t*), 0 ≤ *k* ≤ *p* have closed form solutions. *B*_0_(*t*) is the cumulative baseline hazard and *B*_*k*_(*t*), 1 ≤ *k* ≤ *p* are the excess cumulative hazards at time *t*, which are defined from time 0 to the maximal time *τ* at which the design matrix based on the covariates *Z* and the observed times is full rank [1, 3]. If *Z*_*k*_ is an indicator (0/1) for some *k*, 1 ≤ *k* ≤ *p*, the estimate of *B*_*k*_(*t*) gives the additional cumulative hazard estimate at time *t* for being in the *Z*_*k*_ = 1 group while adjusting for the other covariates. The nonparametric additive hazard model was adjusted for the same covariates as in the semiparametric additive hazard model. Similar Cox-Snell residual plot as in the semiparametric model was generated with the difference that all the residuals for the nonparametric additive model were censored at the maximal time *τ* [3].

Traditional Cox proportional hazard models for the incident detection of oncogenic and any HPV, incorporating the same covariates as previously mentioned, were run for comparison to the additive models. All the statistical analyses were conducted using SAS 9.1.3, and the plots were generated with R 2.9.2. The computer code can be downloaded at https://sites.google.com/site/samxiepage/Additive_Model_Pkg.zip?attredirects=0&d=1.

## 3. Results

The Cox proportional hazard model for the incident detection of oncogenic HPV showed that HIV-positive women with CD4 > 500 had a hazard ratio (HR) 1.62 with 95% confidence interval (CI) 1.31 to 2.00 relative to HIV-negative women. The corresponding HRs and 95% CIs comparing HIV-positive women with CD4 200–500 and CD4 < 200, using HIV-negative women as the reference group, were 2.49 (CI: 2.04–3.03) and 3.82 (CI: 3.01–4.86), respectively. The *P* for trend was calculated by treating HIV/CD4 group as an ordinal variable with four levels (0 to 3) and was highly significant (*P* < 0.0001). In addition, age was negatively associated, and smoking was positively associated, with incident detection of oncogenic HPV. In models of the incident detection of any HPV, the HRs and 95% CIs for HIV-positive women with CD4 > 500, CD4 200–500, and CD4 < 200 were 1.65 (CI: 1.39–1.96), 2.76 (CI: 2.33–3.27), and 3.40 (CI: 2.66–4.34), respectively. The *P* for trend was less than 0.0001. Similar significant factors as in the incident oncogenic HPV were found with the additional findings that African American women had higher incidence of any HPV than Caucasian women, and the number of male sexual partners in the past 6 months was positively associated with the incident detection of any HPV. 

These hazard ratios, however, did not address the absolute number of new HPV infections that would be detected with a decrease in CD4 count. Further, checking of the proportionality assumption for the Cox models shows that the proportionality of hazard function did not hold for the HIV-positive with CD4 < 200 in oncogenic HPV analysis (*P* = 0.046) and for the number of male sexual partner in past 6 months ≥3 in any HPV analysis (*P* = 0.02). For these reasons, we applied the additive hazards regression models to this data set.

The semiparametric additive hazard model for oncogenic HPV was fitted, and the results are given in Table 1. HIV-positive women with CD4 > 500 had an additional hazard of 0.03 than the HIV-negative women, which implies that on average there were 3 additional oncogenic HPV infection cases per 100 HIV-positive women per year with CD4 > 500 compared to HIV-negative women; HIV-positive women with CD4 200–500 had an increase of hazard 0.08; HIV-positive women with CD4 < 200 had an increase of hazard 0.14. All the increases relative to HIV-negative women were statistically significant (*P* < 0.0001), and the increasing trend with respect to HIV/CD4 group was significant with *P* value < 0.0001. The effects of age, race, smoking, and number of male sexual partner in past 6 months agreed with those from the corresponding Cox model.

**Figure 1:**
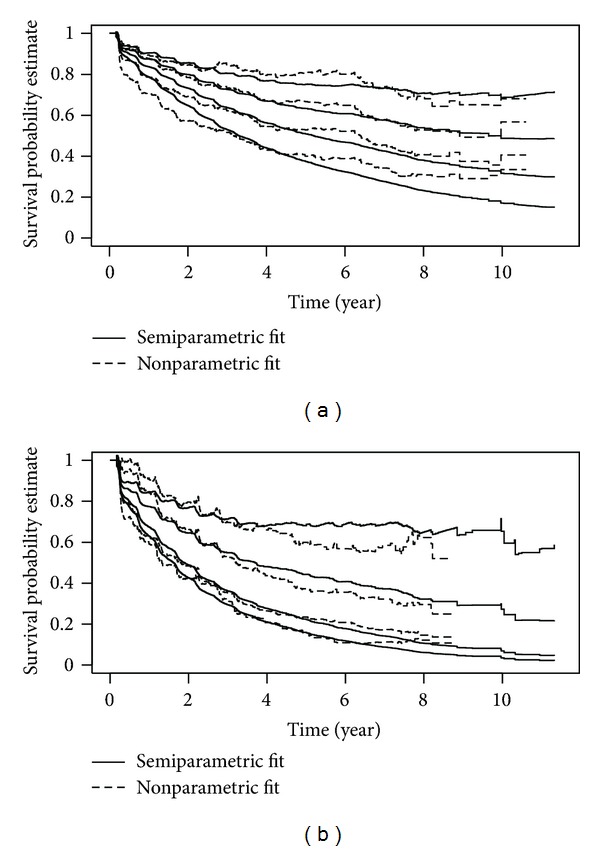
Estimates of survival probabilities of oncogenic HPV and any HPV for the HIV/CD4 strata from semiparametric and nonparametric additive hazard model fitting with the other covariates held at reference values: age < 30, race is white, never smoked, and one male sexual partner in past 6 months: (a) oncogenic HPV; (b) any HPV. From top to bottom for each outcome and each model fit: HIV−, CD4 > 500, CD4: 200–500, and CD4 < 200.

The estimated survival probabilities for the four HIV/CD4 strata adjusted for other covariates from the semiparametric additive model are given in Figure 1(a). It shows that lower CD4 count was associated with increased detection of oncogenic HPV. 

The nonparametric additive hazard model was also fitted to the data. The variables in the nonparametric additive hazard regression model had similar statistical significance to those in the semiparametric additive model and also to those in the Cox proportional hazard regression model with the same covariates. Figure 1(a) shows the estimates of survival probabilities of oncogenic HPV for the four HIV/CD4 groups: $\exp\left(-\hat{B}{\;}_{0}(t)\right)$, $\exp\left(-(\hat{B}{\;}_{0}(t)+\hat{B}{\;}_{1}(t))\right)$, $\exp\left(-(\hat{B}{\;}_{0}(t)+\hat{B}{\;}_{2}(t))\right)$, $\exp\left(-(\hat{B}{\;}_{0}(t)+\hat{B}{\;}_{3}(t))\right)$ for HIV-negative women, HIV-positive women with CD4 > 500, CD4 200–500, and CD4 < 200, respectively, adjusted for other covariates, where $\hat{B}{\;}_{0}(t)$ is the estimated cumulative baseline hazard and $\hat{B}{\;}_{i}\left(t\right),\;\;i=1,2,3$ is the estimated excess cumulative hazard associated for each CD4 stratum. Figure 1(a) shows that the semiparametric model (model (1)) and the nonparametric models (model (2)) in general gave similar estimates on cumulative hazards functions. In particular, the distances between the curves are similar, indicating that these two models gave close estimates of CD4 effect. 

**Figure 2:**
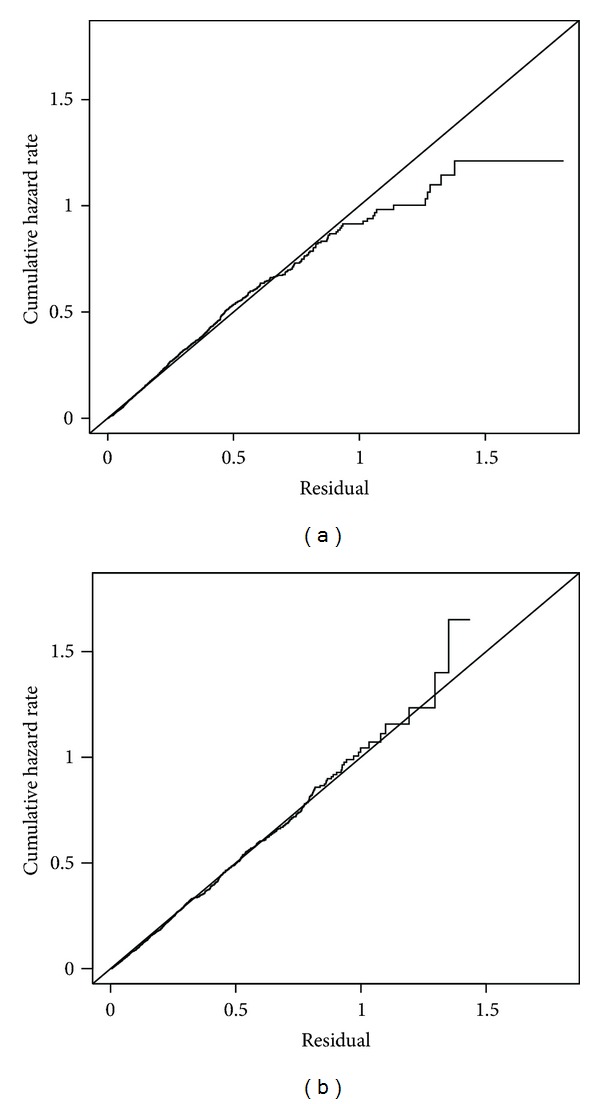
Cox-Snell residual plots for oncogenic HPV models with diagonal reference lines: (a) semiparametric additive model; (b) nonparametric additive model.

In this analysis based on model (2), the estimated survival probability of oncogenic HPV over 5 years of followup among HIV-negative women with an age < 30, of Caucasian race, who were nonsmokers, and had only one male sexual partner in the past 6 months, was 0.80. The corresponding cumulative incidence was 1 − 0.80 = 0.20, which implies that over 5 years of followup 20% of HIV-negative women with the previously mentioned characteristics had at least one positive test for oncogenic HPV; the cumulative incidence rates by 5 years of followup were 0.33, 0.47, and 0.60 for CD4 > 500, CD4 200–500, and CD4 < 200 groups, respectively. Thus, for every 100 women with CD4 < 200, there were 40 more oncogenic HPV infections by year 5 compared to every 100 HIV-negative women, which is a significant increase in number of infections. Both the semiparametric and nonparametric additive hazard models fit the data well based on the Cox-Snell residual plots (Figure 2): the estimated cumulative hazard curves approximately follow the 45 degree lines.

**Figure 3:**
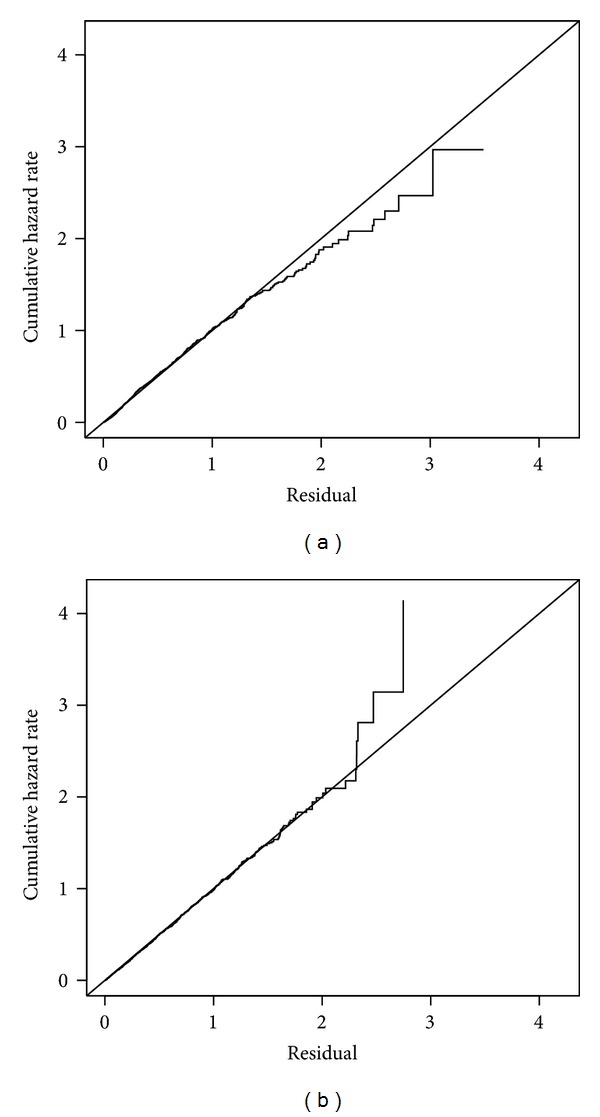
Cox-Snell residual plots for any HPV models with diagonal reference lines: (a) semiparametric additive model; (b) nonparametric additive model.

The same analyses were conducted for any HPV (Table 1). The effect estimates for HIV-positive women with CD4 > 500, CD4 200–500, CD4 < 200 were 0.09, 0.23, 0.30, respectively, with *P* values less than 0.0001 (*P* for trend < 0.0001). From the nonparametric additive model (Figure 1(b)), the difference in survival of any HPV between CD4 200–500 and CD4 < 200 group was not as significant as that for survival of oncogenic HPV. The cumulative incidences of any HPV at 5 years were 0.40, 0.63, 0.77, 0.84 for HIV-negative women, HIV-positive women with CD4 > 500, CD4 200–500, CD4 < 200, respectively. The additive hazard models for any HPV also fit the data well (Figure 3).

## 4. Conclusion

This study applied two types of additive hazard regression models: the semiparametric and the nonparametric additive hazards regression models and a Cox proportional hazard model to the analysis of HPV incidence detection data in HIV-positive and HIV-negative women and contrasted the effect estimates obtained using each statistical approach. All models found highly significant associations between host immune status and risk of incident HPV detection. The semiparametric additive model showed that on average there were an additional 14 oncogenic HPV infection cases per 100 woman-years related to CD4 count < 200 relative to HIV-negative women; and the nonparametric model showed an additional 40 oncogenic HPV infections per 100 women after 5 years of followup.

While as expected the additive models had much lower effect estimates than the Cox model, the two approaches address different questions; that is, the Cox model provides estimates of relative hazard (on a multiplicative scale), whereas the additive hazard models provide approximate estimates for the attributable risk (i.e., the absolute difference in the event rate per unit of change in the exposure variable) under rare event assumption. The attributable risk can be used to determine the absolute increase in number of cases, that is, the number of extra cases of HPV infection that occurred due to the exposure of interest. The relative hazards estimated by Cox models can be especially useful in understanding the magnitude of association, which may be important scientifically; that is, when the baseline hazard of disease is low the absolute number of additional cases related to exposure may be small, but the relative risk can still be strong. However, the absolute risk can be especially useful for public health planning and intervention, when the actual number of additional cases of a disease is of interest. 

We considered a semiparametric and a nonparametric additive hazard models. Comparing to the semiparametric additive hazard regression model, the nonparametric additive hazard model allows the covariate effects to vary over time nonparametrically and thus provides a more robust estimate of the cumulative hazard function than the semiparametric additive hazard model. However, the nonparametric models also use more statistical degrees of freedom. Therefore, if the average covariate effective estimates are of primary interest the semiparametric additive hazard model could be used, but if one wants to examine whether or not some covariate effects are varying over time or the cumulative hazard function (or the cumulative incidence rate) is of primary interest, the nonparametric additive hazard model may be preferred.

We note that the model proposed by Lin and Ying [4] has been extended to include both additive and multiplicative covariate effects [6, 7]. This model may be necessary, for example, when certain covariates in a Cox proportional hazards model satisfy the proportional hazards assumption and others do not. However, the interpretation of this model is not as straightforward as either the Cox model or the additive models. 

In summary, although the theoretical foundation for the additive hazard models is well established and computer codes for fitting these models are available, they have been less often used than other methods of time-to-event analysis. This may partly reflect a degree of unfamiliarity with these models in the general research community. Continued efforts to increase awareness of these statistical methods are needed and should be considered by biostatisticians and epidemiologists involved in teaching the next generation of researchers. 

## Figures and Tables

**Table 1:** Results from semiparametric additive hazard model fitting for oncogenic HPV and any HPV.

	Oncogenic HPV			Any HPV		
	Effect estimate	SE*	*P* value	Effect estimate	SE	*P* value
HIV/CD4 Count						
HIV- (ref^†^)	0			0		
CD4 > 500	0.0343	0.0075	<0.0001	0.0878	0.0159	<0.0001
CD4: 200–500	0.0779	0.0086	<0.0001	0.2264	0.0214	<0.0001
CD4 < 200	0.1395	0.0163	<0.0001	0.2954	0.0427	<0.0001
Age						
<30 (ref)	0			0		
30–34	−0.0213	0.0103	0.04	−0.0472	0.0205	0.02
35–39	−0.0425	0.0097	<0.0001	−0.0547	0.0202	0.01
40–44	−0.0502	0.0106	<0.0001	−0.0282	0.0230	0.22
≥45	−0.0408	0.0137	0.003	−0.0623	0.0274	0.02
Race						
White (ref)	0			0		
Black	0.0140	0.0096	0.15	0.0517	0.0198	0.01
Hispanic	−0.0030	0.0102	0.77	0.0118	0.0212	0.58
Other	−0.0090	0.0191	0.64	0.0661	0.0485	0.17
Smoking						
Never (ref)	0			0		
Former	0.0034	0.0098	0.73	0.0060	0.0212	0.78
Current	0.0224	0.0079	0.005	0.0495	0.0166	0.003
Sex^‡^						
1 (ref)	0			0		
0	0.0003	0.0082	0.97	−0.0255	0.0162	0.12
2	0.0140	0.0122	0.25	0.0556	0.0256	0.03
≥3	0.0158	0.0128	0.22	0.1217	0.0330	0.0002

*Standard error.

^†^Reference category.

^‡^Number of male sexual partner in past 6 months.
